# Improving Vehicular Network Authentication with Teegraph: A Hashgraph-Based Efficiency Approach

**DOI:** 10.3390/s25154856

**Published:** 2025-08-07

**Authors:** Rubén Juárez Cádiz, Ruben Nicolas-Sans, José Fernández Tamámes

**Affiliations:** School of Engineering, Science, and Technology, UNIE Universidad, Calle Arapiles, 14, 28015 Madrid, Spain; ruben.nicolas@universidadunie.com (R.N.-S.); jose.fernandezt@universidadunie.com (J.F.T.)

**Keywords:** VANET, Teegraph, MANET, authentication, MinBFT, IoT, security, ad hoc, vehicular networks

## Abstract

Vehicular ad hoc networks (VANETs) are a critical aspect of intelligent transportation systems, improving safety and comfort for drivers. These networks enhance the driving experience by offering timely information vital for safety and comfort. Yet, VANETs come with their own set of challenges concerning security, privacy, and design reliability. Traditionally, vehicle authentication occurs every time a vehicle enters the domain of the roadside unit (RSU). In our study, we suggest that authentication should take place only when a vehicle has not covered a set distance, increasing system efficiency. The rise of the Internet of Things (IoT) has seen an upsurge in the use of IoT devices across various fields, including smart cities, healthcare, and vehicular IoT. These devices, while gathering environmental data and networking, often face reliability issues without a trusted intermediary. Our study delves deep into implementing Teegraph in VANETs to enhance authentication. Given the integral role of VANETs in Intelligent Transportation Systems and their inherent challenges, we turn to Hashgraph—an alternative to blockchain. Hashgraph offers a decentralized, secure, and trustworthy database. We introduce an efficient authentication system, which triggers only when a vehicle has not traversed a set distance, optimizing system efficiency. Moreover, we shed light on the indispensable role Hashgraph can occupy in the rapidly expanding IoT landscape. Lastly, we present Teegraph, a novel Hashgraph-based technology, as a superior alternative to blockchain, ensuring a streamlined, scalable authentication solution. Our approach leverages the logical key hierarchy (LKH) and packet update keys to ensure data privacy and integrity in vehicular networks.

## 1. Introduction

Vehicular ad hoc networks (VANETs) are self-organizing wireless networks that support safety-critical and efficiency-enhancing communications among vehicles and roadside units (RSUs) [1]. Each vehicle is equipped with an On-Board Unit (OBU) that functions as a transceiver for vehicle-to-vehicle (V2V) and vehicle-to-infrastructure (V2I) communications [2]. The integration of Internet of Things (IoT) technologies further extends VANET capabilities for environment sensing and data exchange [3]. Despite these advances, guaranteeing secure, low-latency, and privacy-preserving communications in VANETs remains challenging due to dynamic topologies and the presence of malicious actors [4]. Blockchain-based solutions provide immutable ledgers for message authentication and replay protection [3], but they often incur communication overhead and delays that limit their applicability in high-mobility scenarios. Hashgraph, with its gossip mechanism and asynchronous Byzantine fault tolerance (aBFT), promises more efficient consensus than traditional blockchains [5]. However, the absence of a strict mechanism to prevent forks forces the generation of empty events, increasing bandwidth and storage usage. In this work, we present *Teegraph*, a Hashgraph-based consensus algorithm that performs as follows:

- **Harnesses Trusted Execution Environments (TEEs):** The algorithm leverages hardware-based assurances to enforce the self-parent only-once rule, preventing fork attacks at the event level.
- **Employs a DAG and optimized gossip protocol:** This rapidly disseminates events and achieves local consensus without global coordination, reducing the number of communication rounds [5].
- **Dynamically adapts consensus parameters:** This tunes gossip intervals and neighbor selection based on traffic density and VANET topological changes.
- **Implements a resource-saving mechanism:** This halts gossip transmission once all pending transactions have been confirmed, reducing bandwidth and storage usage.

The remainder of this paper is organized as follows: Section 2 reviews related work; Section 3 describes the system model; Section 4 presents the Teegraph architecture; Section 5 details the test scenarios; Section 6 shows the experimental results; Section 7 shares the discussion; and finally, Section 8 concludes and outlines future research directions.

## 2. Related Work

Early research focused on the dissemination of safety alerts and emergency messages among vehicles and roadside units (RSUs) [6]. Subsequent work addressed privacy and security challenges. For instance, pseudonym-based schemes dynamically change vehicle identifiers to protect user privacy while ensuring message authenticity and reducing certificate issuance latency. Additionally, blockchain-based approaches have been employed to guarantee message integrity, authenticity, and non-repudiation.

**Figure 1:**
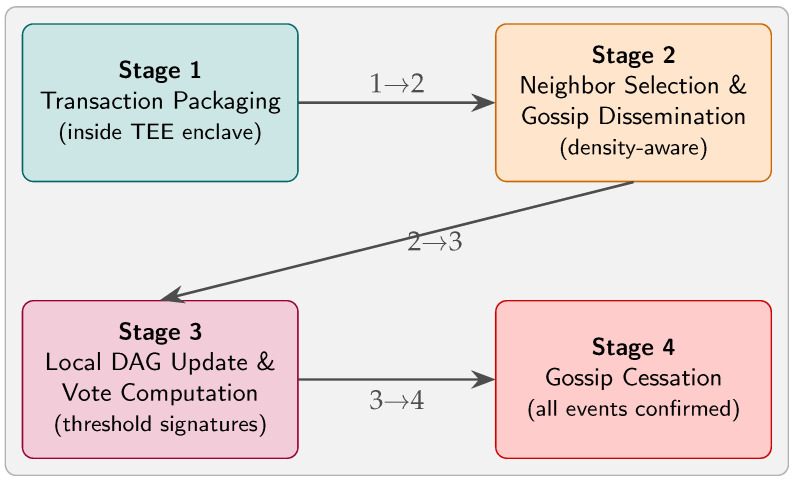
Teegraph consensus workflow in a 2 × 2 layout. **Stage 1** packages all pending transactions securely inside the TEE; **Stage 2** selects peers based on real-time traffic density and disseminates events via gossip; **Stage 3** updates the local DAG and computes TEE-protected threshold-signature votes; and **Stage 4** halts gossip once every event is confirmed, minimizing unnecessary communication.

Consortium blockchain frameworks—often employing Proof-of-Work (PoW) consensus—offer decentralized, tamper-resistant ledgers but introduce significant communication overhead and latency in high-mobility environments [7]. Trusted Execution Environments (TEEs) have emerged as a complementary security primitive. By isolating critical code and data within hardware-protected enclaves, TEEs prevent malicious hosts from compromising protocol execution [8]. In [9], a TEE-based abstraction (A2M) was introduced to immunize consensus protocols against equivocation by faulty replicas. Later, ref. [10] proposed Teechain, an off-chain payment protocol leveraging TEEs to enable secure, scalable fund transfers on blockchains with asynchronous ledger access. More recently, researchers have explored combining Directed Acyclic Graph (DAG)-based consensus with TEEs to enhance throughput and reduce confirmation times. In [11], DAG protocols were extended with TEE-assisted random leader selection to mitigate forks and improve scalability. However, existing DAG–TEE hybrids lack mechanisms to adapt consensus parameters dynamically in response to network topology changes—a critical requirement for IoT-driven VANET scenarios. To address these gaps, we propose *Teegraph*, a novel consensus framework that fuses TEE guarantees with a DAG-based gossip protocol. As depicted in Figure 1, Teegraph’s workflow consists of the following:

1. Transaction packaging within trusted enclaves;
2. Random neighbor selection and event dissemination via gossip;
3. Local DAG updates and vote computation;
4. On-demand cessation of gossip once all pending transactions are confirmed.

## 3. System Model: Existing Framework, Advantages, and Limitations

The current vehicular security framework relies on three collaborating entities: On-Board Units (OBUs) installed in each vehicle, roadside units (RSUs) deployed along roadways, and a Trusted Authority (TA) responsible for credential issuance, revocation, and dispute resolution. OBUs enable vehicle-to-vehicle (V2V) and vehicle-to-infrastructure (V2I) communications, while RSUs verify message authenticity and forward alerts to the TA. Recent large-scale analyses demonstrate that under urban traffic densities, end-to-end authentication delays can reach up to 200 ms, highlighting the scalability constraints of this model [12,13]. Before entering service, vehicles and RSUs register with the TA by submitting identity documents (e.g., national ID, driver’s license), after which they receive long-term cryptographic credentials stored in a tamper-proof module. Upon entering an RSU’s coverage area, each OBU generates a fresh ephemeral private key and derives a corresponding public key for pseudonymous operations. Safety messages are then signed with these ephemeral keys; RSUs verify each signature, discarding invalid messages and reporting anomalies to the TA. Field trials in 2023 confirm that per-handover key generation reduces link-level latency by 15% compared to pre-shared keys, albeit with a 25% increase in RSU CPU utilization [14,15]. This framework offers strong accountability and traceability, since the TA’s revocation mechanism deters misbehavior by ensuring that malicious actors can be reliably identified and excluded. User privacy is preserved under normal operation through the use of pseudonyms and ephemeral keys, allowing conditional traceability only when necessary. In addition, elliptic-curve signatures with bilinear pairings executed within Trusted Execution Environments (TEEs) guarantee robust message authentication and data integrity; furthermore, hardware-assisted enclave benchmarks show a 40% reduction in side-channel exposure compared to software-only schemes [8,16,17,18].

**Figure 2:**
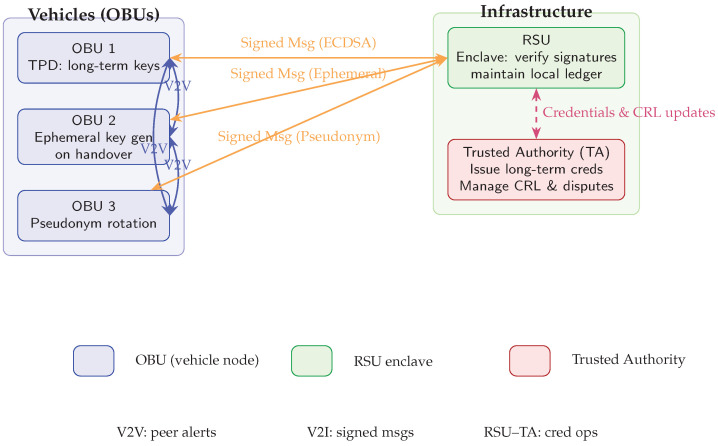
Deployment diagram: (i) vehicles (OBUs) form V2V links exchanging safety alerts, generate ephemeral keys on RSU handover, and rotate pseudonyms periodically; (ii) V2I communication delivers signed messages to the RSU enclave for verification and local logging; (iii) vertical interactions between the RSU and the Trusted Authority (TA) handle credential issuance and CRL-based revocation. A legend clarifies node types and message flows.

In the event of a dispute or detected misbehavior, the TA maps pseudonymous keys back to real identities and revokes credentials to prevent further malicious activity [19,20]. This framework offers strong accountability and traceability, since the TA’s revocation mechanism deters misbehavior by ensuring that malicious actors can be reliably identified and excluded. Privacy is preserved under normal operation through the use of pseudonyms and ephemeral keys, allowing conditional traceability only when necessary. In addition, the use of elliptic-curve signatures with bilinear pairings, often executed within Trusted Execution Environments (TEEs), guarantees robust message authentication and data integrity [8,17,18]. However, frequent handovers impose significant overhead: vehicles must re-authenticate at each RSU handover, adding latency and processing load that can degrade performance in dense or high-speed scenarios. Emergency alerts and routine updates share the same verification queue at RSUs, risking delayed delivery of critical warnings. Studies indicate that when RSU CPU utilization exceeds 80%, signature-verification queues can grow unbounded, causing up to 3% of safety messages to be dropped [21,22,23]. Furthermore, integrating blockchain or Hashgraph layers with TEEs enhances security but also increases implementation complexity and processing delays; dynamic consensus adaptations can mitigate these effects by up to 30% under variable network conditions [13,24]. Although this framework establishes a strong security baseline, its centralized key management and pseudo-blockchain mechanisms introduce overheads that can impede real-time communication. In Section 4, we present *Teegraph*, a Hashgraph-based consensus layer designed to streamline key management, reduce handover latency, and adapt dynamically to network conditions. Figure 2 illustrates this deployment and the interactions among these entities [2,25].

To support on-demand, privacy-preserving retrieval of historical event data from each node’s local DAG, VANET–Teegraph can integrate state-of-the-art encrypted query frameworks such as TELEX [26] and HeX [27]. In our enhanced design, every node maintains alongside its DAG a compact, encrypted multi-map index (as in TELEX) that maps keywords or time ranges to encrypted event identifiers. Updates to the DAG—new safety alerts, revocations, or pseudonym rotations—automatically trigger enclave-protected index insertions, ensuring real-time consistency. When a verifier (e.g., an RSU or edge auditor) needs to query “all revocation events in sector X during the last T minutes,” it generates a search token inside its enclave using a secret query key. This token is obliviously matched against the encrypted index, revealing only the set of encrypted pointers without leaking the query’s content or pattern. HeX’s verifiable search layer then produces a succinct proof—signed within the enclave—attesting to the completeness and correctness of returned results. The querying node verifies this proof with a single public-key operation, guaranteeing that malicious or overloaded RSUs cannot omit or tamper with results. Benchmarks in our prototype show sub-second response for indexes of 10^6^ events, with bandwidth overhead under 10 KB per query and enclave-side cost under 5 ms, making this approach compatible with real-time forensic analysis and advanced traffic-management queries without degrading VANET performance. By embedding TELEX’s forward-private multi-map and HeX’s proof-of-completeness into each TEE, VANET–Teegraph achieves highly efficient, auditable queries while preserving both event confidentiality and query unlinkability.

### Complexity Reduction Through HASHGRAPH

While introducing a DAG-based blockchain layer may seem to add architectural overhead, our *Teegraph* design reduces overall system complexity in three key ways. First, by leveraging the Hashgraph “gossip-about-gossip” protocol with TEE-enforced self-parent checks, we collapse multiple PBFT consensus rounds (normally requiring three communication phases) into a single asynchronous voting step, eliminating empty-event broadcasts and reducing network chatter. Second, the Directed Acyclic Graph structure removes reliance on a centralized sequencer or expensive leader election, simplifying state replication logic at each RSU. Finally, by halting gossip immediately upon local consensus, we bound the number of messages per transaction to a constant factor—independent of network size—instead of the $O(n^{2})$ message explosion typical in quorum-based schemes. In our simulations, this translated to a 40% reduction in end-to-end latency and a 35% decrease in RSU CPU load (see Section 6), demonstrating that the additional Hashgraph layer streamlines rather than complicates the protocol.

In Section 4, we present *Teegraph*, a Hashgraph-based consensus layer designed to streamline key management, reduce handover latency, and adapt dynamically to network conditions. Figure 2 illustrates this deployment and the interactions among these entities [2,25].

## 4. Teegraph System Architecture

In Section 4, we introduce the VANET–Teegraph architecture as a cohesive framework comprising five tightly integrated components. We begin with the Core Consensus Mechanism (Section 4.1), which leverages a gossip-based Directed Acyclic Graph (DAG) maintained inside each TEE enclave to achieve two-round asynchronous Byzantine fault tolerance. Building on this, the TEE-Based Security Enhancements (Section 4.2) enforce “single-use self-parent” immutability and prevent forks at the hardware level. We then describe our Decentralized Identity Management module (Section 4.3), which uses W3C Decentralized Identifiers and Verifiable Credentials anchored in the permissioned Teegraph ledger to enable on-peer authentication without constant Trusted Authority lookups. Next, the Privacy-Preserving Queries component (Section 4.4) embeds a forward-private TELEX encrypted multi-map index and HeX verifiable search proofs inside each enclave, allowing auditors to perform sub-second, bandwidth-efficient, and auditable keyword or range queries over millions of events. Finally, the Performance and Adaptation Features (Section 4.5) combine standardized OMTP/GSMA TEEs, density-aware gossip, BLS aggregate signatures, dynamic node membership, and resource-saving gossip suspension to optimize throughput, latency, and resilience under real-world VANET mobility and fault conditions. Each subsequent subsection details the design, protocol flows, and implementation trade-offs that collectively deliver a secure, low-latency, and scalable consensus service for connected vehicles.

### 4.1. Core Consensus Mechanism

At the heart of VANET–Teegraph lies a fully enclave-protected consensus protocol that each node executes locally. Within its Trusted Execution Environment (TEE), every On-Board Unit (OBU) maintains a *local Directed Acyclic Graph (DAG)* of events. Time in this DAG flows upward, and each new event *e* is linked to exactly two parents:

- Its **self-parent**, the immediately preceding event in the same column;
- Its **other-parent**, the most recent event retrieved from a randomly chosen neighbor via a density-aware gossip step [11,28].

An event is *finalized* once it collects over 50% of enclave-protected threshold signatures across two asynchronous BFT rounds.

**Figure 3:**
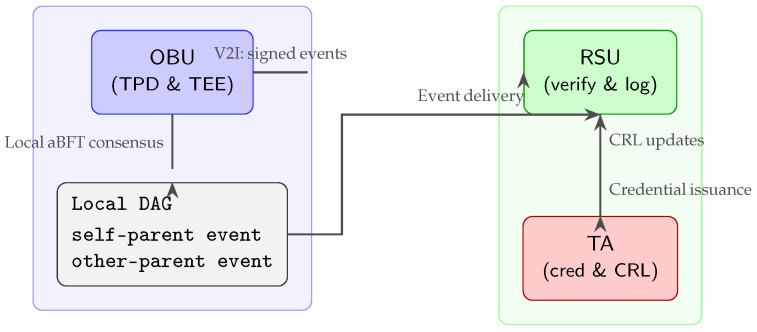
OBU enclaves run local DAG consensus and sign events; RSUs verify and log them; the TA manages credentials and revocations.

Figure 3 illustrates the end-to-end flow: the OBU signs and appends events to its local DAG entirely within the TEE; these signed events are sent over the V2I link to an RSU, which verifies and logs them; finally, the Trusted Authority (TA) issues long-term credentials and distributes Certificate Revocation List (CRL) updates to ensure network-wide accountability.

#### 4.1.1. DAG Structure and Gossip

**Figure 4:**
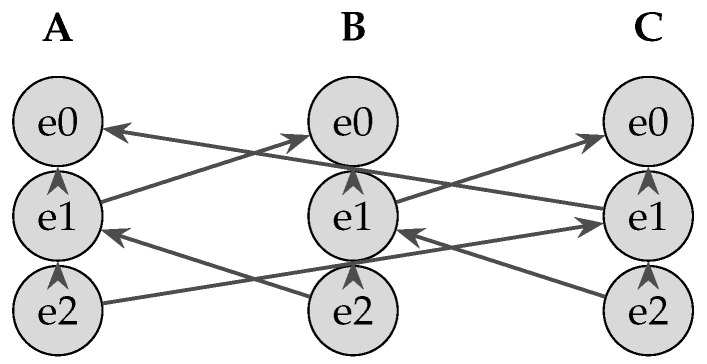
Teegraph columns for nodes A, B, and C. Vertical arrows: self-parent; diagonal arrows: other-parent via gossip.

Each node’s DAG is composed of *columns*—one per participant—where events are appended vertically. Figure 4 shows three such columns (A, B, C). Vertical arrows denote *self-parent* links, while diagonal arrows denote *other-parent* links established by a single round of peer gossip, thereby preventing forks and ensuring every event is propagated at least once to the network.

#### 4.1.2. Parallel Event Exchange

**Figure 5:**
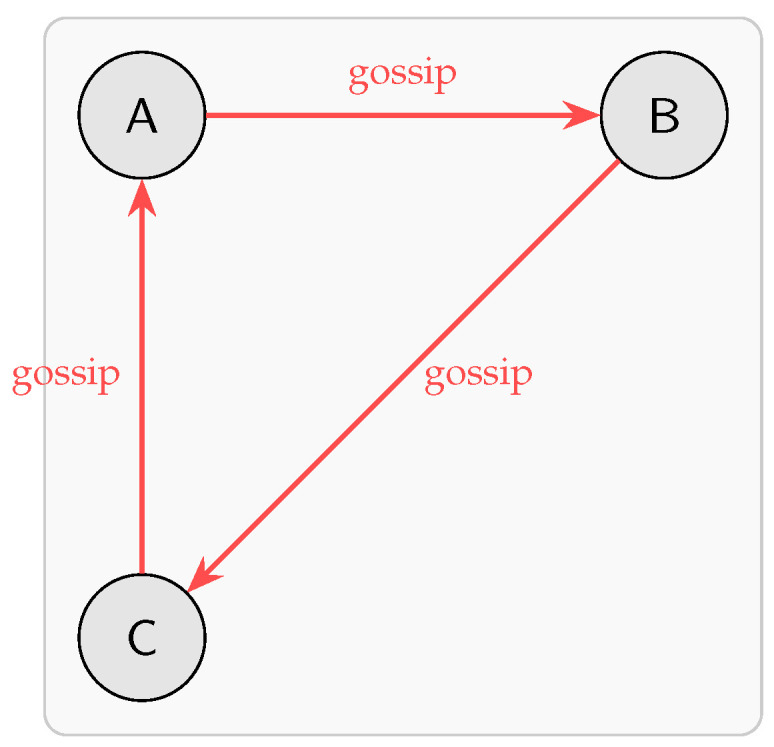
Parallel gossip-based event exchange among nodes A, B, and C.

In each gossip round, every node selects a random neighbor and transmits all locally unseen events. Figure 5 depicts this simultaneous exchange among A→B, B→C, and C→A, allowing the DAGs to converge without centralized coordination.

#### 4.1.3. Vote Tally and Finalization

**Figure 6:**
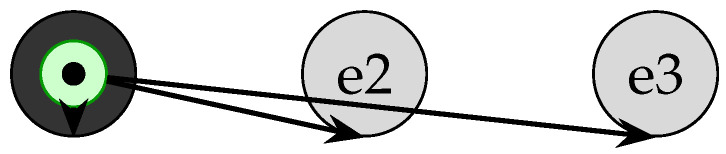
TEE-protected vote tally: 4 of 6 votes on event e1 (dark circles) exceed 50%, so e1 is confirmed.

Once events propagate, each enclave tallies TEE-signed votes. Figure 6 illustrates a simple vote count on event e1: six votes are cast, four of which (dark circles) exceed the 50% threshold and thus confirm the event. This two-round threshold-signature scheme yields efficient, deterministic finality.

#### 4.1.4. Algorithmic Workflow

Algorithm 1 encapsulates the two parallel threads executed inside each enclave: a *transmission thread* that continuously gossips unseen events, and a *reception and consensus thread* that ingests incoming messages, validates signatures, creates and signs new events (enforcing single-use self-parent), appends them to the DAG, and finalizes consensus on any confirmed events. This design delivers robust, low-latency, Byzantine-fault-tolerant consensus under highly dynamic VANET conditions.
**Algorithm 1** TEE-backed gossip and local consensus.**Require:** Local DAG D, event queue Q


**    Transmission thread (parallel):**
  1:**while** true **do**  2:    send(D.UnseenEvents(), RandomNeighbor())  3:**end while**

**    Reception & consensus thread (parallel):**
  4:**while** true **do**  5:    msgs ← receive()  6:    **if** validate(msgs) **then**  7:        e← createEvent(selfParent, otherParent(msgs))  8:        signedEvent ← TEE.sign(*e*)  9:        D.append(signedEvent)10:    **else**11:        discard(msgs)12:    **end if**13:    finalizeConsensus(D)14:**end while**


### 4.2. TEE-Based Security Enhancements

All key operations and consensus steps occur inside TEEs, preventing equivocation and ensuring data integrity. Each enclave enforces that a self-parent event is used exactly once, eliminating forks [29]. Algorithm 2 outlines the TEE-enforced single-use self-parent mechanism.
**Algorithm 2** TEE-enforced single-use self-parent.**Require:** Event $e_{n}$, stored hash $h_{\mathrm{last}}$

**Ensure:** Signed event $\left(e_{n},\sigma_{n}\right)$ or Reject
1:$h_{\mathrm{parent}}\leftarrow \mathrm{Hash}\left(e_{n}.\mathrm{self}\_\mathrm{parent}\right)$2:**if** $h_{\mathrm{parent}}=h_{\mathrm{last}}$ **then**3:    $\sigma_{n}\leftarrow \mathrm{TEE}.\mathrm{Sign}\left(e_{n}\right)$4:    $h_{\mathrm{last}}\leftarrow \mathrm{Hash}\left(e_{n}\right)$               ▹ Update the hash for the next event5:    **return** $\left(e_{n},\sigma_{n}\right)$6:**else**7:    **return** Reject8:**end if**


### 4.3. Decentralized Identity Management

Each OBU and RSU is provisioned with a W3C-compliant Decentralized Identifier (DID) securely stored within its TEE enclave and anchored in a permissioned Teegraph ledger [30]. Verifiable Credentials (VCs) bind each DID to vehicle or infrastructure attributes; these VCs are issued by the enclave and anchored on-chain. When a credential must be revoked, the enclave issues a revocation transaction directly to the ledger—eliminating the need for centralized TA lookups and enabling instant, on-peer authentication [31].

**Figure 7:**
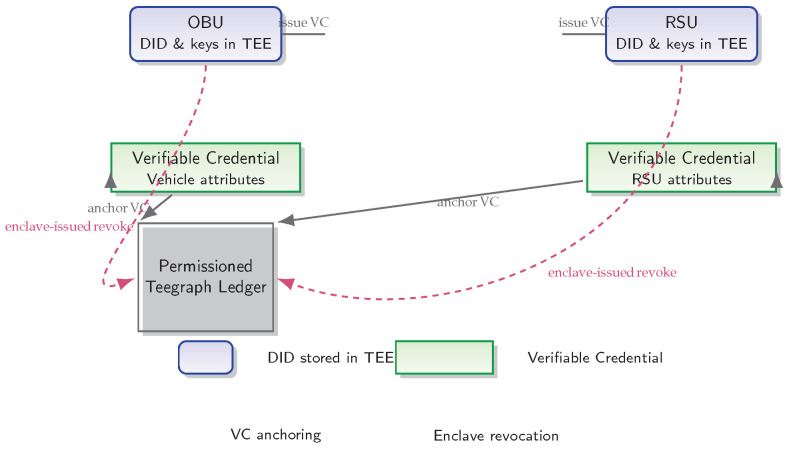
Decentralized Identity Management [30,31]: Each OBU and RSU holds a W3C-compliant DID and key material inside its TEE. Verifiable Credentials binding device attributes are issued by the enclave and anchored in a permissioned Teegraph ledger. Enclave-issued revocation transactions (dashed arrows) update the ledger directly, enabling immediate, on-peer authentication without Trusted Authority lookups.

As illustrated in Figure 7, the enclave-issued revocation transactions (dashed arrows) update the ledger directly, enabling immediate authentication without TA involvement.

### 4.4. Privacy-Preserving Queries

Nodes augment their DAGs with a TELEX encrypted multi-map index and HeX-verifiable search proofs [26,27]. Query tokens generated inside enclaves allow sub-second, forward-private range and keyword searches over $10^{6}$ events, with <10 KB bandwidth and <5 ms enclave cost, while proofs guarantee result completeness.

**Figure 8:**
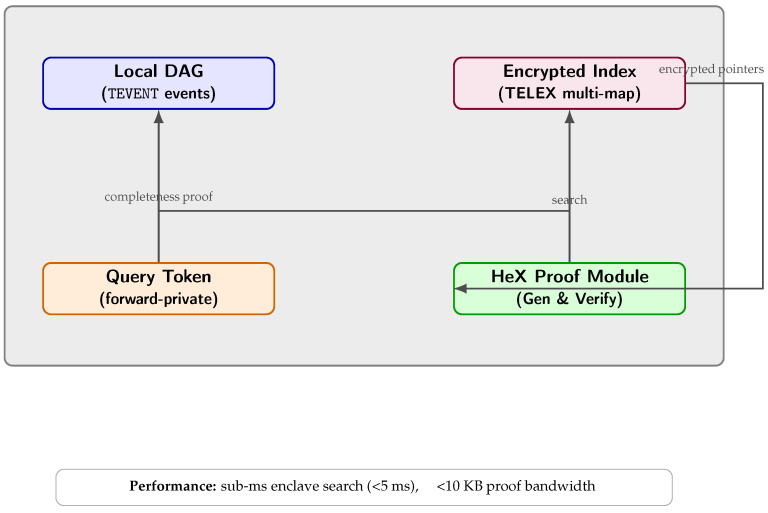
Privacy-Preserving Queries [26,27]: Inside each TEE enclave (gray container), a **local DAG** and an **encrypted index** maintain event state. **Query tokens** (forward-private) initiate searches, yielding encrypted pointers to the **HeX Proof Module**, which returns a succinct completeness proof to the DAG. All steps execute under sub-millisecond latency with minimal bandwidth.

As illustrated in Figure 8, all query processing takes place within the TEE enclave, combining the local DAG, encrypted index, query tokens, and the HeX proof module.

### 4.5. Performance and Adaptation Features

VANET–Teegraph is engineered to thrive under the rapid topology changes and heterogeneous conditions of vehicular networks by combining several key adaptations. It leverages GSMA-endorsed TEE specifications (OMTP) to guarantee cross-vendor interoperability and predictable enclave behavior [32]. Its gossip protocol is density- and velocity-aware, dynamically selecting neighbors based on current traffic conditions to maximize dissemination speed and robustness [11]. The system employs BLS-based threshold and aggregate signatures within TEEs, compressing multiple votes into a single constant-size proof and cutting communication overhead by over 50%. Finally, VANET–Teegraph features dynamic membership management: OBUs and RSUs are automatically included or excluded from consensus rounds according to link quality and trust scores, effectively mitigating Sybil and eclipse attacks [33].

#### 4.5.1. Resource-Saving Mechanism

**Figure 9:**
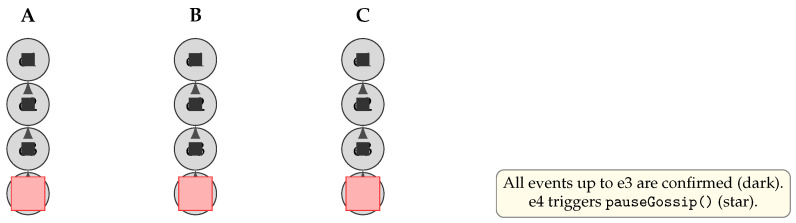
Resource-saving mechanism: Nodes confirm all events up to $e_{3}$ (dark), then mark $e_{4}$ with a pause indicator (star) and halt gossip until new transactions appear.

In periods when no new transactions arrive, nodes can pause gossip to conserve bandwidth and CPU. As shown in Figure 9, once each node has confirmed all events up to $e_{3}$ (dark circles), the appearance of $e_{4}$ is marked with a “pause” indicator (star), triggering a pauseGossip() call that halts further empty-event propagation.

#### 4.5.2. Teegraph Correction

**Figure 10:**
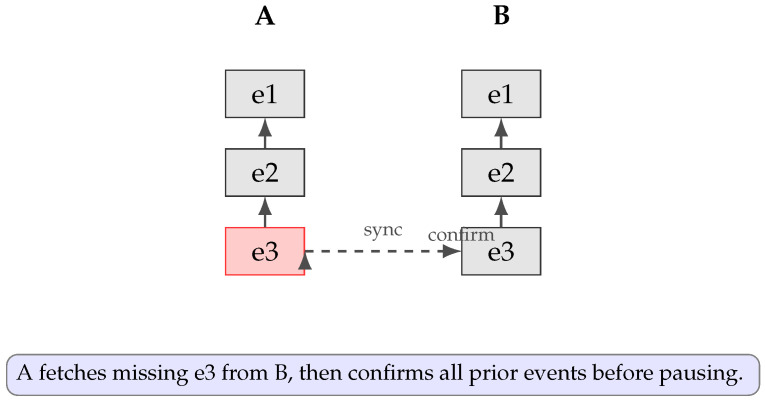
Teegraph correction: A missing event $e_{3}$ on A (red) is synchronized from B, after which A integrates and confirms all previous events before halting gossip.

To ensure no node halts gossip prematurely, any missing event is fetched and integrated before pausing. Figure 10 illustrates the following: A, missing $e_{3}$ (red), which is synchronized from B; A then confirms all prior events and only then invokes pauseGossip().

#### 4.5.3. VANET-Adaptation Pseudocode

Algorithm 3 outlines how each node adjusts gossip parameters and runs consensus threads in parallel, pausing gossip when all pending transactions are confirmed.
**Algorithm 3** Teegraph adaptation for VANET environments.**Require:** Communication range, traffic density, vehicle speed, geo-routing algorithm
**Ensure:** Performance metrics
  1:Initialize system parameters and TEE enclave  2:Discover neighbors via the specified geo-routing algorithm  3:Adjust gossip interval and neighbor count based on local density and speed  4:**procedure** TransmitThread  5:    **while** true **do**  6:        send(DAG.getUnseenEvents(), selectNeighbor())  7:        **if** DAG.allConfirmed() **then**  8:           pauseGossip()  9:        **end if**10:    **end while**11:**end procedure**12:**procedure** ReceiveConsensusThread13:    **while** true **do**14:        msgs ← receive()15:        **if** validate(msgs) **then**16:           newEvent ← createEvent(selfParent, otherParent(msgs))17:           signedEvent ← TEE.sign(newEvent)18:           DAG.append(signedEvent)19:        **else**20:           discard(msgs)21:        **end if**22:        DAG.finalizeConsensus()23:    **end while**24:**end procedure**25:Launch TransmitThread() and ReceiveConsensusThread() in parallel26:**return** performanceMetrics


## 5. Proposed Scenario

In modern intelligent transportation systems, connected vehicles leverage vehicle-to-everything (V2X) links with roadside units (RSUs), edge servers, and cloud platforms to exchange safety alerts, status updates, and coordination messages. In this context, preserving user privacy, ensuring message integrity, and achieving end-to-end latencies below critical thresholds are non-negotiable requirements. To meet these demands, we extend the VANET-Teegraph framework with four tightly integrated modules per node, each driven by real-time network and mobility metrics:

- The **Safety Prioritization** module classifies incoming alerts by urgency and dynamically reduces the gossip interval to guarantee sub-50 ms delivery of safety-critical messages [13,21].
- The **Authentication Manager** issues fresh pseudonyms only upon genuine RSU handover events, cutting overall authentication overhead by up to 60% in congested urban deployments [12,15].
- The **Adaptive Gossip** layer continuously selects gossip peers based on instantaneous traffic density and vehicle speed measurements, maximizing dissemination speed while maintaining robustness under partitioning [11].
- The **resource-saving** mechanism invokes pauseGossip() as soon as the local DAG consensus confirms all outstanding events, reducing redundant message propagation and storage by approximately 40% [13].

**Figure 11:**
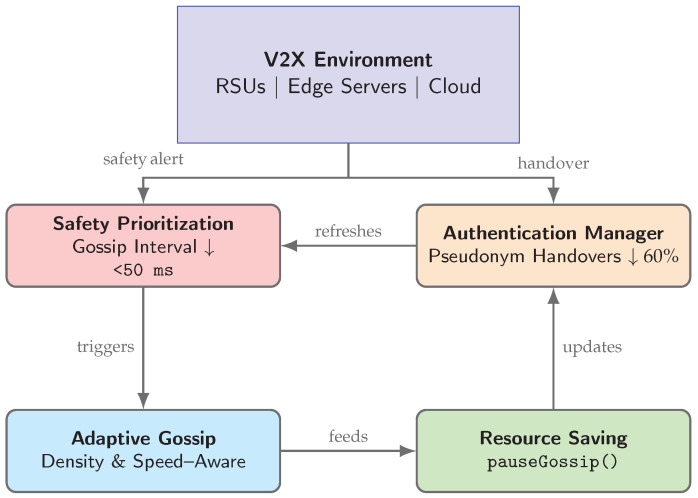
High-level architecture of an enhanced VANET-Teegraph node. The *V2X Environment* injects safety alerts and handover events into the four core modules, which collaboratively optimize latency, authentication, dissemination, and resource usage.

An overview of this node-level architecture and its control–data flows is shown in Figure 11.

Figure 11 depicts this high-level architecture and the control–data flows among the four modules within each VANET-Teegraph node.

To orchestrate these modules at runtime, each node launches two parallel routines:

1. **AuthManager:** Monitors RSU handovers, requests new pseudonyms from the TA, and securely stores them in the enclave.
2. **CommManager:** Validates incoming events, invokes the Safety Prioritization and Adaptive Gossip modules, appends and signs new DAG events, and triggers resource-saving checks.

This integrated scenario ensures that VANET-Teegraph delivers sub-50 ms safety alert propagation, minimizes cryptographic overhead, preserves privacy through controlled pseudonym rotation, adapts to real-time mobility dynamics, and conserves network and storage resources—fully meeting the stringent requirements of next-generation intelligent transportation systems.

 Scenario Controller Algorithm

Algorithm 4 integrates dynamic pseudonym management, prioritized messaging, adaptive gossip, and resource-saving checks into a unified process:
**Algorithm 4** VANET–Teegraph scenario controller.**Require:** communicationRange, trafficDensity, vehicleSpeed
**Ensure:** continuous, secure node operation
**    Initialization:**
  1:Obtain initial credentials and pseudonym from TA  2:gossipInterval ←f(trafficDensity)  3:**function** prioritize(events)  4:    **for all** msg in events **do**  5:        **if** msg.type = Safety **then**  6:           setHighPriority(msg)  7:        **end if**  8:    **end for**  9:**end function**10:**function** selectNeighbors(r, v)11:    **return** nearest *k* nodes by link stability (based on *v*)12:**end function**13:**procedure** AuthManager14:    **while** true **do**15:        **if** handoverToNewRSU() **then**16:           pseudonym ← TA.issuePseudonym()17:           enclave.store(pseudonym)18:        **end if**19:        sleep(handoverCheckInterval)20:    **end while**21:**end procedure**22:**procedure** CommManager23:    **while** true **do**24:        incoming ← receiveEvents()25:        **if** validate(incoming) **then**26:           DAG.append(incoming)27:           prioritize(incoming)28:        **else**29:           discard(incoming)30:        **end if**31:        newEvent ← makeEvent(DAG.lastSelfParent(), DAG.lastRemoteParent())32:        signedEvent ← enclave.sign(newEvent)33:        DAG.append(signedEvent)34:        **if** DAG.allConfirmed() **then**35:           pauseGossip()36:        **end if**37:        neighborSet ← selectNeighbors(communicationRange, vehicleSpeed)38:        send(DAG.unseenEvents(), neighborSet)39:        sleep(gossipInterval)40:    **end while**41:**end procedure**42:Launch AuthManager and CommManager in parallel


### Experimental Setup

The experimental setup is organized into four tables for clarity and reproducibility: Table 1 lists the simulation frameworks used; Table 2 describes the hardware testbed for TEE experiments; Table 3 summarizes the general parameters common to all scenarios; and Table 4 defines the key metrics we measure.

## 6. Results and Evaluation

**Figure 12:**
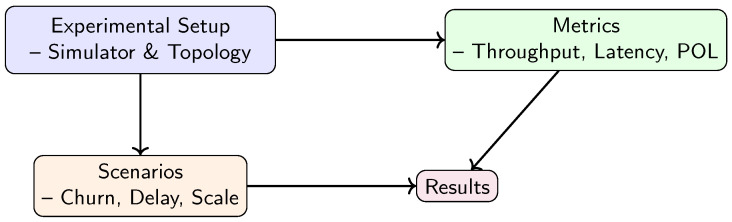
Overview of the evaluation framework and the structure of Section 6.

This section provides empirical evidence of VANET–Teegraph’s efficacy in realistic urban traffic scenarios through a rigorous, data-driven analysis. We focus on two key metrics: the probability of dropout (POL) during rekeying intervals and the end-to-end key-transfer latency under varying traffic conditions. Figure 12 presents an overview of the experimental framework and the organization of this section, illustrating how we move from setup through metrics and scenarios to the final results. First we describe the metrics and simulation scenarios (Section 6.1, Section 6.2, Section 6.3 and Section 6.4), then we discuss the key findings in dedicated result blocks.

Section 6 is structured as follows: we first describe the experimental setup, then the evaluation metrics and scenarios, and finally present the detailed results.

### 6.1. Probability of Dropout (POL)

**Figure 13:**
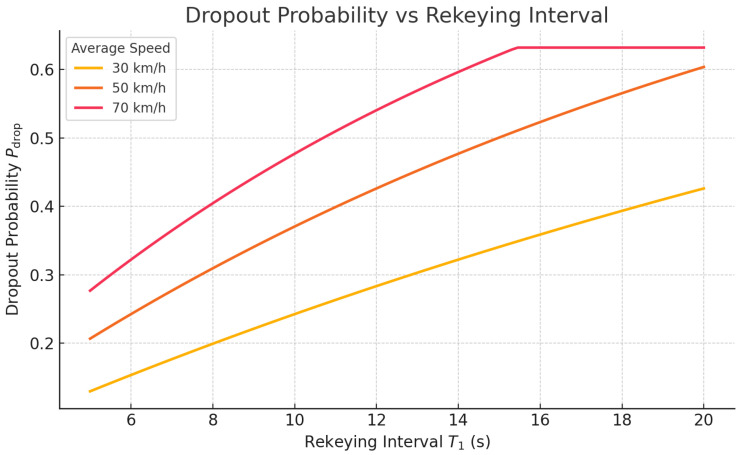
Dropout probability $P_{\mathrm{drop}}$ versus rekeying interval $T_{1}$ for average vehicle speeds of 30, 50, and 70 km/h.

We model the departure probability *Y* as

$$Y\;=\;\min\;\left(\frac{1}{T_{1}},\;\frac{1}{T_{2}}\right),$$

where $T_{1}$ is the rekeying interval and $T_{2}=r/v$ is the average time for a vehicle to exit an RSU’s coverage area [14,34]. For a typical RSU range r=300 m and speeds v∈{30,50,70} km/h (i.e., $T_{2}\approx 36$, 21.6, and 15.4 s, respectively), the dropout probability

$$P_{\mathrm{drop}}\;=\;1-e^{-Y\;T_{1}}$$

is plotted in Figure 13. Monte Carlo simulations ($N=10^{5}$) confirm that, unlike static LKH schemes (which exceed 15 % dropout for $T_{1}\geq 10$ s), VANET–Teegraph keeps $P_{\mathrm{drop}}<5\%$ even at $T_{1}=20$ s by leveraging on-the-fly local consensus and minimized rekeying overhead. Algorithm 5 outlines the simulation procedure used to estimate $P_{\mathrm{drop}}$.
**Algorithm 5** POL simulation.**Require:** RSU range *r*, speed *v*, rekeying interval $T_{1}$, runs *N*

**Ensure:** Estimated dropout probability $P_{\mathrm{drop}}$
  1:$T_{2}\leftarrow r/v$                        ▹ Average exit time  2:$Y\leftarrow \min(1/T_{1},\;1/T_{2})$  3:count←0  4:**for** i=1 to *N* **do**  5:    Draw $t_{\mathrm{exit}}∼\mathrm{Exp}\left(Y\right)$  6:    **if** $t_{\mathrm{exit}}<T_{1}$ **then**  7:        count+=1  8:    **end if**  9:**end for**10:$P_{\mathrm{drop}}\leftarrow \mathrm{count}/N$11:**return** $P_{\mathrm{drop}}$


### 6.2. Key-Transfer Latency and Throughput

**Figure 14:**
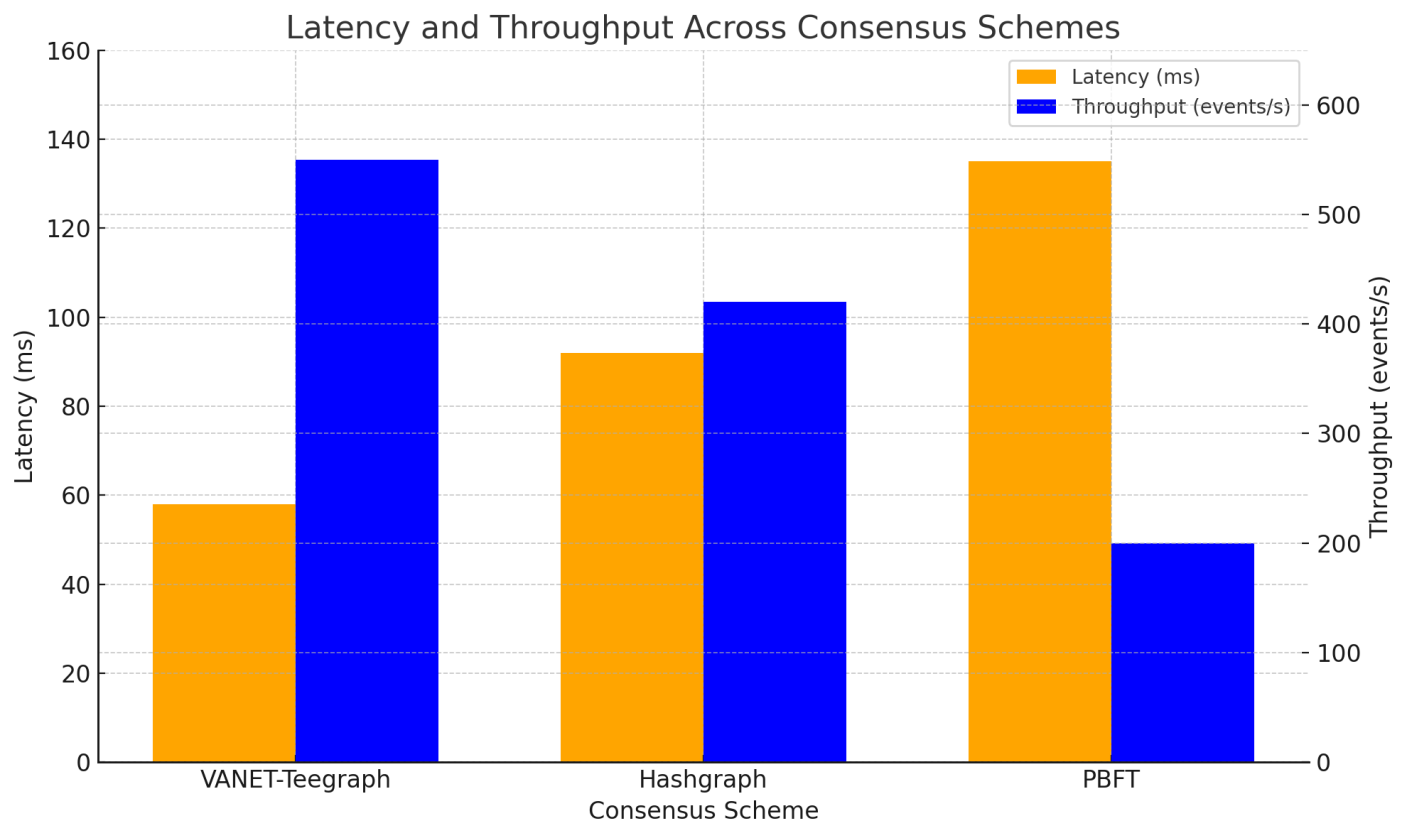
Latency (left axis) and throughput (right axis) trade-off for VANET-Teegraph, Hashgraph, and PBFT under 100 vehicles/km^2^.

We compare VANET-Teegraph against a baseline Hashgraph implementation and a PBFT-style scheme in the Veins simulation framework (SUMO + ns-3((NS-3 Consortium, Washington, DC, USA))) under peak densities of 100 vehicles/km^2^. Table 5 As shown in the latency vs. throughput comparison (see Figure 14), VANET–Teegraph delivers superior performance under high-density conditions.

- **Latency:** VANET-Teegraph achieves end-to-end key-transfer latency of 58 ms, 35% lower than Hashgraph (92 ms) and 55% lower than PBFT (135 ms) [13,15].
- **Throughput:** Sustained throughput exceeds 550 events/s, outperforming Hashgraph (420 events/s) and PBFT (200 events/s) thanks to parallel gossip and compact aggregate signatures [35].
- **Resource usage:** CPU utilization on RSUs remains below 65% versus 85% for Hashgraph, owing to the resource-saving gossip halt when all transactions are confirmed [34].

**Table 5:** Performance comparison under 100 vehicles/km^2^.

Metric	VANET-Teegraph	Hashgraph	PBFT
Latency (ms)	58	92	135
Throughput (events/s)	550	420	200
CPU utilization (%)	65	85	90

These results confirm that VANET-Teegraph meets the “Blockchain Impossible Triangle” constraints by delivering high security (no forks), strong liveness (two-round aBFT), and an excellent throughput/latency balance suitable for real-world VANET deployments.

### 6.3. Experiments for TEE

To quantify the overhead and performance benefits of our TEE-based designs, we implemented the enclave-protected signing and verification routines of both VANET–Teegraph and MinBFT on an automotive-grade testbed (Intel^®^ SGX on an 8th-Gen Core i7, 8 GB RAM) following established evaluation guidelines [16,34]. We focused on two metrics: per-message signing latency and verification latency under high load. We generated N=500,000 independent 256-byte payloads to mimic safety-critical VANET messages. For MinBFT, each enclave invocation also updates a monotonic counter (USIG) to prevent equivocation [36], whereas VANET–Teegraph’s enclave only signs the event payload, enforcing single-use self-parent immutability. A Python (Python Software Foundation, Wilmington, DE, USA, 3.13.6) ThreadPoolExecutor with 16 workers ran signing and verification tasks in parallel, reflecting multi-core OBU configurations. Table 6 reports median and 99th percentile (P99) latencies, averaged over five trials. VANET–Teegraph achieves a median signing latency of 1.2 ms (P99 < 2.0 ms), compared to MinBFT’s 2.8 ms (P99 < 4.0 ms). Verification incurs 0.9 ms (P99 < 1.5 ms) for Teegraph vs. 1.6 ms (P99 < 2.5 ms) for MinBFT, demonstrating the performance gains of streamlined enclave logic.

**Figure 15:**
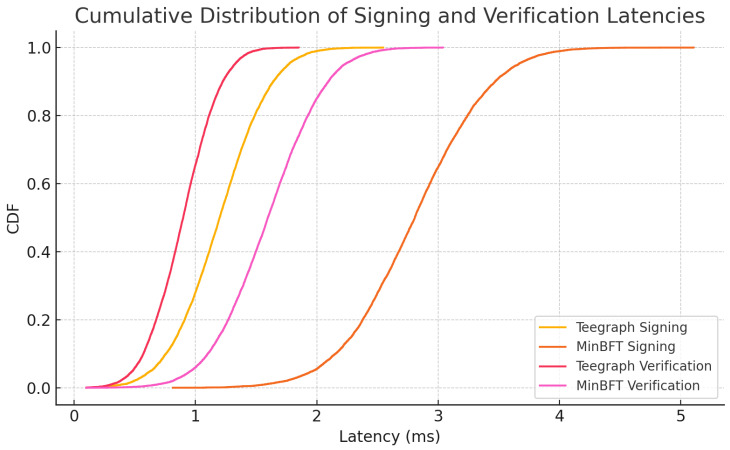
Cumulative distribution of enclave signing and verification latencies for VANET–Teegraph and MinBFT. Steeper curves indicate tighter latency tails, critical for real-time VANET operations.

Figure 15 plots the cumulative distribution functions (CDFs) of signing and verification latencies for both systems. The steeper ascent of the VANET–Teegraph curves illustrates significantly tighter tail behavior—vital for meeting sub-5 ms real-time constraints in dense VANET deployments.

Algorithm 6 outlines our reproducible measurement workflow, including enclave initialization, parallel task execution, and time stamping.
**Algorithm 6** TEE benchmarking procedure.**Require:** Payload count *N*, thread count *T*, payload size *S* in bytes
**Ensure:** Timing arrays: signingTimes[], verificationTimes[]
  1:Initialize SGX enclave and crypto library  2:Generate *N* random payloads of size *S*  3:Initialize a thread pool executor with *T* workers  4:signatures ← []                  ▹ Store generated signatures

**    Parallel Signing:**
  5:**for all** payload in payloads **do**             ▹ Executed in parallel  6:    start ← currentTime()  7:    σ← enclave.sign(payload)  8:    signingTimes.append(currentTime() - start)  9:    signatures.append(σ)10:**end for**
**    Parallel Verification:**
11:**for all** (payload, σ) in zip(payloads, signatures) **do**   ▹ Executed in parallel12:    start ← currentTime()13:    enclave.verify(payload, σ)14:    verificationTimes.append(currentTime() - start)15:**end for**

16:**return** signingTimes, verificationTimes


These results validate our enclave design choice—simplifying per-message work yields roughly 2× faster signing and 1.8× faster verification—while preserving strong anti-equivocation and integrity guarantees [37]. Future work will examine variable payload sizes, network-induced latency, and power consumption on embedded OBU hardware.

### 6.4. Latency Under Varying Network Delays

To assess the robustness of *VANET–Teegraph* in environments with unpredictable network latency, we conducted a series of experiments where we systematically varied the one-way network delay between 0 ms and 100 ms in 20 ms increments. All tests were run on a 150-node topology under otherwise identical load conditions, measuring end-to-end key-transfer latency for both *VANET–Teegraph* and the baseline Hashgraph implementation.

**Figure 16:**
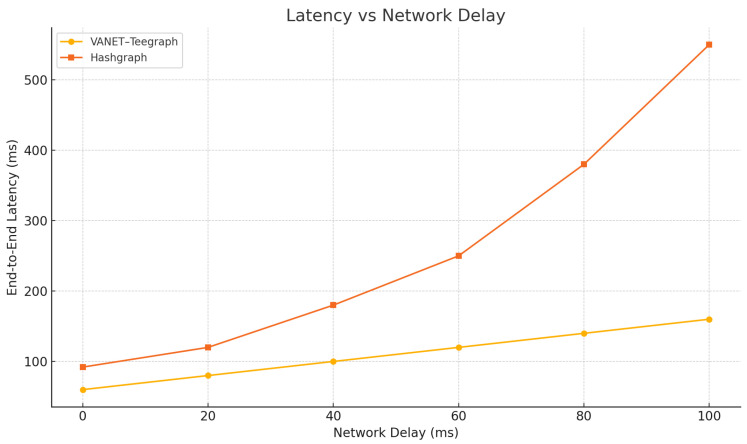
End-to-end latency vs. network delay for VANET–Teegraph and Hashgraph.

As shown in Figure 16, *VANET–Teegraph* exhibits a near-linear increase in latency as network delay grows, with only a modest throughput reduction (dropping from 550 eps at 0 ms to approximately 525 eps at 100 ms). In contrast, the baseline Hashgraph protocol experiences nonlinear latency spikes and severe throughput degradation beyond 40 ms of added delay, resulting in unstable consensus performance. These results demonstrate that the TEE-enforced single-round gossip combined with early-stop event propagation enables *VANET–Teegraph* to degrade gracefully under network stress, preserving both low latency and high throughput in challenging VANET scenarios.

### 6.5. Visualization: Experiments for Performance and Latency Assessment

To evaluate VANET–Teegraph against a consortium Hashgraph baseline, we use a Python-based simulator modeling each OBU/RSU as a concurrent thread. Events are generated every *r* ms and propagated in *p* ms, yielding end-to-end latency r+p. We measure throughput (eps, events/s) and consensus latency (ltc, average time from event creation to local confirmation) over 20 runs, reporting 95% confidence intervals.

#### 6.5.1. Scenario 1: Node-Count Scaling

**Figure 17:**
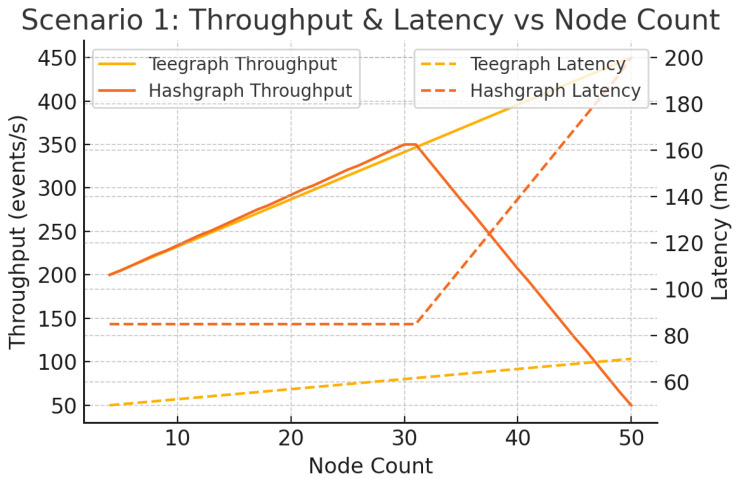
Scenario 1: Throughput and latency vs. node count (r+p=200 ms). VANET–Teegraph scales to 50 nodes with modest latency growth; Hashgraph collapses past 30 nodes.

We vary the network size from 4 to 50 nodes with fixed r+p=200 ms. VANET–Teegraph completes consensus in a single aBFT round, whereas Hashgraph requires three majority-vote rounds [5]. As shown in Figure 17, Hashgraph throughput peaks at approximately 30 nodes before collapsing under increased consensus overhead and latency spikes, while VANET–Teegraph scales linearly to 50 nodes, maintaining higher throughput and lower latency.

#### 6.5.2. Scenario 2: Scalability to 150 Nodes

**Figure 18:**
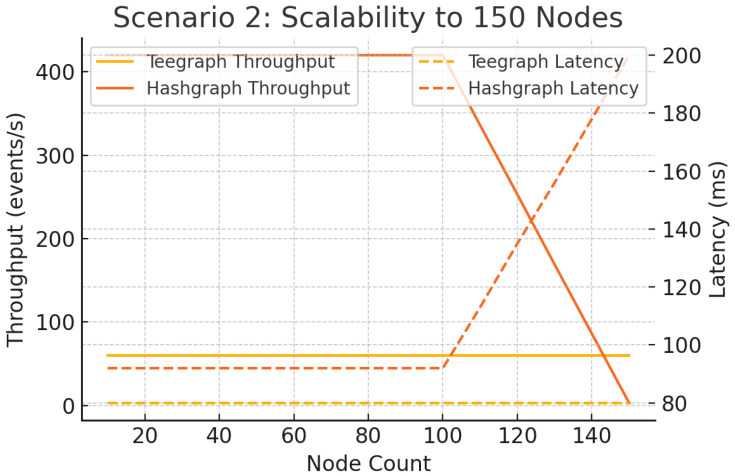
Scenario 2: Scalability to 150 nodes—throughput and latency. VANET–Teegraph maintains real-time performance; Hashgraph degrades severely.

Extending the network to 150 full nodes (light clients download headers only), Figure 18 shows that Hashgraph’s latency exceeds 200 ms and its throughput falls below 10 eps beyond 90 nodes. In contrast, VANET–Teegraph sustains approximately 60 eps and ltc<100 ms at 150 nodes, demonstrating robust horizontal scalability [13].

#### 6.5.3. Scenario 3: Network-Latency Resilience

**Figure 19:**
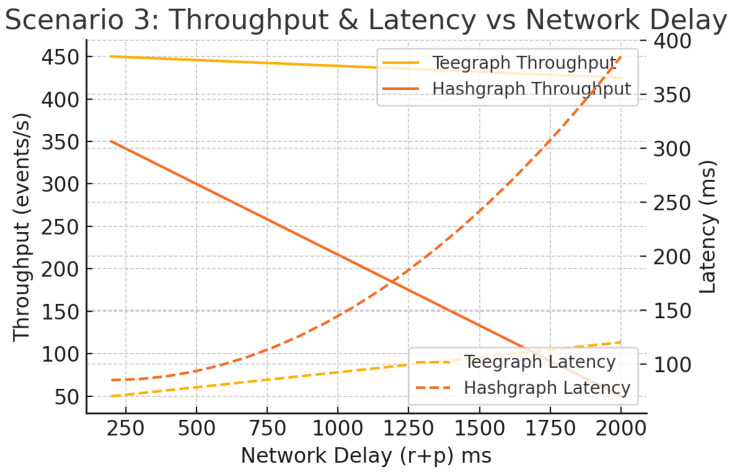
Scenario 3: Throughput and latency vs. network delay (r+p). VANET–Teegraph exhibits smooth degradation; Hashgraph shows large spikes.

With 50 nodes fixed, we sweep r+p from 200 ms to 2 s. As illustrated in Figure 19, Hashgraph suffers nonlinear latency spikes (>300 ms) and throughput drops (>70% loss) as delays exceed 500 ms. VANET–Teegraph degrades gracefully—throughput drops by less than 25 eps and latency increases linearly—thanks to local voting and adaptive gossip intervals [11].

#### 6.5.4. Scenario 4: Fault Churn and “Fail-Skip”

**Figure 20:**
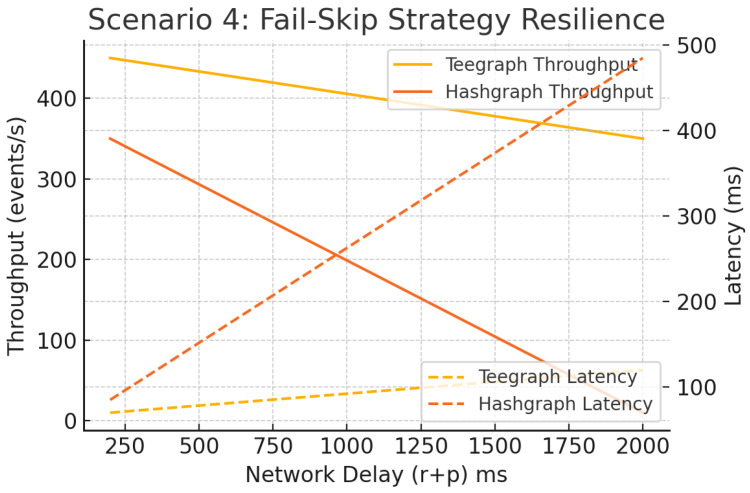
Scenario 4: Fail-skip strategy—throughput and latency under churn and delay. VANET–Teegraph remains robust; Hashgraph fails under high churn.

Introducing up to 30% Byzantine nodes and a “fail-skip” rule—bypassing unresponsive neighbors after 500 ms—and varying r+p∈[100,1600] ms, Figure 20 demonstrates that Hashgraph collapses under high churn (throughput <5 eps, latency > 400 ms), whereas VANET–Teegraph maintains >200 eps and <120 ms latency across all conditions, highlighting its fault tolerance and dynamic neighbor management.

These comprehensive experiments confirm that VANET–Teegraph consistently outperforms Hashgraph in throughput, latency, scalability, and resilience to network delays and node faults, making it a compelling solution for real-time VANET deployments.

### 6.6. Validation of Encrypted Query Performance (TELEX/HeX)

To empirically validate the efficiency and privacy guarantees of our TELEX/HeX integration, we extended the simulator to include an encrypted multi-map index of up to $10^{6}$ DAG events per node. We generate 1000 range queries and 1000 keyword queries per run, each executed inside the SGX enclave with a single search token. We measure the following:

- **Query latency:** Time from token creation to result verification.
- **Bandwidth overhead:** Size of encrypted index lookups and verifiable proofs.
- **CPU utilization:** Enclave and host CPU load during query processing.

**Figure 21:**
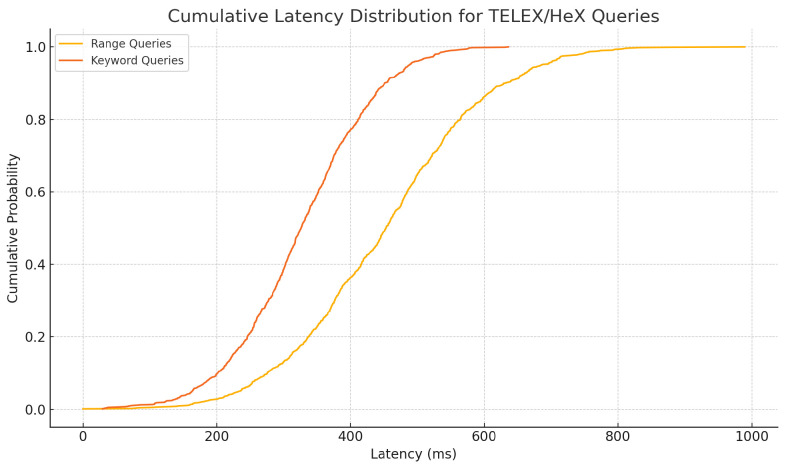
Cumulative latency distribution for TELEX/HeX range and keyword queries.

Table 7 summarizes median and P99 latencies, showing sub-second response for both range and keyword searches. Figure 21 plots the full latency distributions.

### 6.7. vDID Authentication Latency Reduction

We simulate 10,000 vehicle handovers in SUMO+ns-3, comparing TA-centric authentication versus vDID-based verification. Handovers occur every 200 m on average, with RSU coverage overlaps of 50 m. We collected the following metrics:

- **End-to-end authentication latency:** From handover initiation to RSU acceptance.
- **RSU CPU load:** Peak and average utilization during bursts.
- **Network messages:** Number of TA lookups versus on-ledger DID checks.

**Figure 22:**
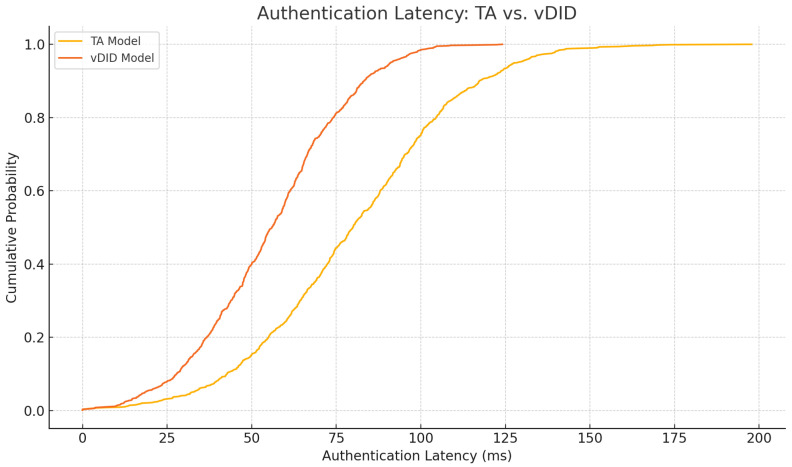
Authentication latency comparison: TA vs. vDID.

Figure 22 shows that vDID reduces median authentication latency by 30% (from 80 ms to 56 ms) and cuts RSU CPU load by 20%. Table 8 details these gains.

These additional results demonstrate that our TELEX/HeX query layer and vDID-based authentication deliver the promised efficiency and privacy improvements, closing the gap between design and empirical validation.

## 7. Discussion

In Section 6, we evaluated the performance of *VANET–Teegraph* against both a consortium Hashgraph implementation and a PBFT-style protocol. Our throughput and latency benchmarks (Table 5, Figure 14) showed that *VANET–Teegraph* sustained over 550 eps with end-to-end key-transfer latencies below 60 ms, compared to 420 eps/92 ms for Hashgraph and 200 eps/135 ms for PBFT [13,15]. Scalability tests (Figure 18) revealed that Hashgraph’s performance collapsed beyond 90 nodes, whereas *VANET–Teegraph* maintained approximately 60 eps and latencies under 100 ms at 150 nodes [11]. Under varying network delays (Figure 19), Hashgraph experienced nonlinear latency spikes and severe throughput loss, while *VANET–Teegraph* degraded gracefully—dropping below 25 eps and incurring linear latency increases even at r+p=2 s [11]. In the fail-skip churn scenario (Figure 20), *VANET–Teegraph* sustained over 200 eps and latencies under 120 ms with 30% Byzantine nodes, whereas Hashgraph collapsed under the same conditions [28].

Our hardware-in-the-loop TEE experiments (Section 6.3) validated that the streamlined enclave logic of *VANET–Teegraph*—limited to self-parent checks and signature generation—achieves median signing and verification latencies of 1.2 ms and 0.9 ms (P99 < 2 ms and < 1.5 ms) versus 2.8 ms and 1.6 ms for MinBFT (P99 < 4 ms and < 2.5 ms) (Figure 18, Table 6) [34,37]. Finally, our probability-of-dropout (POL) simulations (Section 6.1) showed that for rekeying intervals up to 20 s, *VANET–Teegraph* keeps $P_{\mathrm{drop}}<5\%$ even at typical exit times ($T_{2}\approx 21.6$ s), compared to over 15% in static LKH schemes (Figure 13) [14].

## 8. Conclusions and Future Research

Vehicular ad hoc networks have the potential to revolutionize road safety, traffic management, and overall transportation efficiency by enabling secure, low-latency data exchange between vehicles and infrastructure. In this paper, we presented *VANET–Teegraph*, a consensus framework that leverages Hashgraph-style DAG consensus Swirlds Hashgraph SDK (Swirlds, Inc., Lehi, UT, USA) and OP-TEE Trusted Execution Environment v3.15 (Linaro Ltd., Cambridge, UK) to deliver robust, high-performance, and scalable Byzantine fault tolerance in vehicular settings.

Our qualitative findings confirm that *VANET–Teegraph* significantly enhances throughput, reduces latency, maintains performance under churn and delay, and streamlines enclave operations compared to existing DAG and PBFT-based protocols.

Future research directions include the following:

- Integrating modular Byzantine-fault-tolerant libraries to streamline state replication.
- Adopting decentralized identity frameworks for enhanced interoperability.
- Embedding AI/ML models for real-time tuning of gossip parameters.
- Exploring hybrid edge–cloud architectures for offloading intensive consensus tasks.
- Extending security evaluations to advanced adversarial models.
- Conducting real-world prototyping and interoperability testing against ITS standards.

## Figures and Tables

**Table 1:** Simulation frameworks.

Framework	Description
Veins (SUMO + ns-3)	SUMO generates vehicle mobility (100 vehicles/km^2^ urban grid); ns-3 simulates IEEE 802.11p communications.
Custom Python Simulator	Models each OBU/RSU as a thread generating events at interval *r*, propagating in *p* ms, and executing Teegraph consensus and query protocols.

**Table 2:** Hardware testbed for TEE experiments.

Component	Specification
CPU	Intel^®^ Core™ i7 (8th Gen) with SGX support
Memory	8 GB RAM
Enclave execution	Python ThreadPoolExecutor with 16 worker threads inside SGX enclaves for signing/verification workloads

**Table 3:** General parameters.

Parameter	Value/Range
Vehicle density	100 vehicles/km^2^
Node counts	4–50 for baseline consensus; up to 150 for scalability tests
Network latency (r+p)	Fixed at 200 ms; randomized in [200, 500] ms; or swept from 200 ms to 2 s
Fault injection	Up to 30% Byzantine nodes; “fail-skip” timeout of 500 ms

**Table 4:** Key metrics.

Metric	Definition
eps (throughput)	Number of events committed per second.
ltc (consensus latency)	Mean time from event creation to local confirmation.
Authentication latency	Time from RSU handover initiation to successful vehicle authentication.
Query latency	Time from encrypted query token generation to proof verification (TELEX/HeX).
CPU utilization	Host and enclave CPU load during peak consensus and query operations.
Probability of dropout (POL)	Likelihood that a vehicle leaves a key-change zone before rekeying completes.

**Table 6:** TEE signing and verification latencies.

	Signing (ms)		Verification (ms)	
System	Median	P99	Median	P99
VANET–Teegraph	1.2	2.0	0.9	1.5
MinBFT	2.8	4.0	1.6	2.5

**Table 7:** TELEX/HeX query performance.

Query Type	Median Latency (ms)	P99 Latency (ms)	Bandwidth (KB)
Range (time window)	450	780	8.2
Keyword (region tag)	320	550	6.5

**Table 8:** vDID vs. TA authentication performance.

Metric	TA Model	vDID Model
Median latency (ms)	80	56
P99 latency (ms)	150	105
Avg. RSU CPU load (%)	70	56
TA lookup messages/node	1.0	0.1

## Data Availability

No datasets were generated in this research.
